# Collectanea Medica: Consisting of Anecdotes, Facts, Extracts, Illustrations, &c.

**Published:** 1822-10

**Authors:** 


### COLLECTANEA MEDICA:

CONSISTING OF

### ANECDOTES, FACTS, EXTRACTS, ILLUSTRATIONS, &c.

*Relating to the History or the Art of Medicine, and the Collateral Sciences.*

---

Floriferis, ut apes, in saltibus omnia libant,

Omnia nos, itidem, depascimur aurea dicta.

---

### Art. I.—*Case of Stricture of the Rectum, successfully treated by an Operation.* By Dr. Horatio Gates Jameson, of Baltimore.

Miss C. M., about five years since, had a tumor which gradually grew from the rectum till it obtained considerable size, and projected about two inches. She applied to a medical gentleman of this city, who removed it by ligature. The parts soon healed up, but immediately afterwards she laboured under great difficulty in passing the alvine evacuations. The obstruction increased rapidly, and was soon attended with severe pain, and inability to evacuate the bowels without taking physic. Her sufferings she represents as having been very great. She consulted a surgeon of this city, who attempted to relieve her by the use of bougies of different sizes. The use of these soon increased her distress, and, instead of using them of an increased size, as he wished her to do, she was compelled to diminish the size of the bougies, till she found it impracticable to use them of any size.

During an interval of nearly five years, she has had the advice of several highly respectable physicians of this city in succession, who all pronounced it a callous tumor, as well as the surgeon above mentioned. She has long been in a very deplorable condition; and cannot, for some years, have a stool without taking medicine, and, even with the aid of purgatives, the act of passing the fæces is attended with great pain. Extreme pain, constipation, and symptomatic fever, often have seriously threatened her life.

On passing the finger into the rectum, I found the sphincter muscle free from disease, but disposed to contract closely upon the finger; not, however, with any great degree of force. About an inch up the rectum I found an obstruction, having, at first, precisely the feel of a callous tumor, of considerable size. After a more thorough examination, and causing the patient to change the situation of the parts, by pressing down forcibly, while my finger was still introduced, I discovered that what had been considered a tumor was really but a membranous partition of the rectum. Continuing the examination, I discovered that this partition had been formed by the rectum falling down in folds, as we sometimes see in subjects who have died of chronic diarrhœa, and that in this state they must have become inflamed and grown together, leaving a small opening, not in the centre of the partition or septum, but near its pubic side. This opening greatly resembled the os tincæ. It was much thickened and callous, so that it might readily deceive one into the belief of its being a solid tumor. This difficulty was increased by the circumstance that, when the patient strained down, or any fæces lodged behind the septum, the opening was turned up horizontally towards the pubis, and the septum became very tense.

This situation of the parts rendered it almost impossible for the fæces to pass through the opening. Great quantities of muco-purulent discharges have long existed, and have tended to impair her strength. The parts affected are exquisitely tender.

Having satisfied myself that this was the nature of the case, and having represented to the patient that an operation might be practised with good hopes of relieving her; that we had something to fear from hemorrhage, and from peritoneal inflammation; but, upon the whole, I thought the danger would not be great, if she would determine on conforming strictly to my directions after the operation; she determined to submit to any thing I thought proper to try for her recovery from a situation so truly deplorable.

In the presence of my friends, Drs. Murphy and Martin, I operated in the following manner, 22d September, 1821:—I passed my left fore-finger into the rectum, and placed the end against the opening through the septum, but could not pass it through; keeping the finger here, as a guide, firmly pressed against the opening, I conveyed with the right hand a button-pointed bistouri along the finger. I carefully directed the point of the knife with the end of the left fore-finger into the opening. Being satisfied that the end of the knife had passed some distance into the opening or ring of the septum, I cut freely down towards the sacrum by drawing the knife towards me; endeavouring at the same time to direct its movements with the left fore-finger. I thus readily divided the septum down to its termination, and found the rectum perfectly relieved and quite capacious. But, to my great surprise, I discovered, by passing the finger upwards, that another partition existed precisely similar to the first, and about an inch higher up. This I treated in the same manner, and with the same result; removing, by these simple incisions, an obstruction which had tormented the patient for years, and often endangered her life.

Notwithstanding the incisions were made with great freedom, there was very little hemorrhage; my hands were scarcely soiled. A long piece of compressed sponge was now introduced, and the patient, having taken fifty drops of laudanum, was put to bed, and a T bandage applied to retain the sponge in its place. The patient was advised to take fifty drops of laudanum in the evening, with a view of restraining the bowels, as well as to obviate irritation and pain. I had taken care to have the intestines well emptied the night before the operation.

*September 23d.*—Informed that some hemorrhage occurred yesterday soon after I left the patient; she was greatly alarmed. Dr. Murphy saw her, and quieted her fears, and the hemorrhage soon ceased; patient in all might, according to the doctor's computation, have lost fourteen ounces of blood. She had considerable pain at night, and, in a fit of petulant rashness, took upwards of an ounce of laudanum. This brought on high fever, sick stomach, great thirst. The patient drank largely of cold water in the morning, and brought on violent vomiting. She has not had any inclination in the parts to pass off the sponge. I bled her freely, and directed effervescing draughts.

*Sept. 24th.*—Has been reasonably comfortable since yesterday morning. I removed the sponge, without difficulty, this morning: the parts are extremely sore; the rectum quite spacious. Has little or no fever; advised her to refrain from every kind of solid food, and to take a portion of calcined magnesia, to which she has been accustomed.

*Sept. 25th.*—The patient was attacked with vomiting and fever after my visit: was bled and took effervescing draughts, which relieved her perfectly, and I find her doing well this morning. Advised her to take a dose of oil, the magnesia not having acted.—Afternoon: fever has returned, with severe vomiting. I bled her about twelve ounces. In the act of vomiting, this afternoon, she had an involuntary, painful, and copious stool, the first free evacuation she had had for five years.

It seems unnecessary to pursue this case further in detail. There was no difficulty from hemorrhage; nor was there, at any time, any soreness of the abdomen to excite uneasiness in regard to peritoneal inflammation. She was affected, for several days, with fever and sick stomach. Most of these unpleasant symptoms, doubtless, arose from the improper use of laudanum, and from other little improprieties in diet and drinks, which the patient practised.

In about four weeks the parts were healed and the patient restored to health and comfort, although she had a pretty severe bilious attack after I discontinued my attendance.—(*American Medical Recorder*, April 1822.)

---

Art. II.—*Cases illustrative of the Remedial Powers of Prussiate of Iron, in Intermitting and Remitting Fevers.* Communicated by William Zollickoffer, M.D. of Middleburg, Frederick co. Maryland; Honorary Member of the Society of Natural Sciences of St. Gall, Switzerland, &c.

The prussiate of iron possesses the following advantages over the cinchona, as a remedy in intermittent and remittent fevers:

First. It is void of taste, and may therefore be much more readily given to children than the pulv. cinchonæ, which to some is extremely unpleasant.

Secondly. The dose is much smaller, being from four to six grains twice or thrice a-day, or every three hours; while bark, to be effectual, must be given in much larger doses.

Thirdly. In its effects as a remedy calculated to prevent the recurrence of future paroxysms, it is more certain, prompt, and effectual, than the justly celebrated cort. cinchona.

Fourthly. A patient treated with this article will recover from the influence of intermitting and remitting fevers, in the generality of cases, in much less time than is usual in cases treated with bark. In making use of the prussiate of iron as a remedy, care must be taken to select that which is of a very dark blue, approaching to black, having a shining coppery fracture, and adhering firmly to the tongue.

The following cases are adduced in evidence of its efficacy in the diseases mentioned:

Case I.—On the 4th of August, I was called to visit Mr. P—'s lady, who, upon examination, I found to be labouring under the tertian form of intermittent fever. This lady had, a few days previous to my visit, taken an emetic, which was succeeded by a dose of the sub. mur. hyd., and shortly after followed by a small dose of ol. ricini. Under these circumstances, I thought it advisable, inasmuch as the stomach and bowels had been freely evacuated, to prescribe the use of the prussiate of iron, which I directed to be taken morning and evening in a little sugar and water. I left sixteen grains, divided into four doses. She commenced taking the first powder on the morning intervening the disease, and had taken three powders before the time in which she expected the return of the paroxysm; but no symptoms of the disease occurred.

Case II.—August the 7th. Upon this day the case of a child came under my care. The disease was of the quotidian type, under which it had been labouring for eight days. I left a small dose of mur. hyd. mit., which operated *sursum et deorsum* freely; after which I ordered one powder of the prussiate of iron to be given every three hours during the apyrexia. The child had taken but four powders, when the affection disappeared; and it remained hearty for four weeks.

Case III.—August the 8th. I was called upon to visit Miss H—n, who I found affected with the intermittent fever. I left her a dose of sub. mur. hyd. cum. jalapa, which I directed to be taken in a small quantum of syrup. simp. and its operation to be facilitated by drinking any simple tea; after which I ordered one powder of the prussiate of iron, containing four grains each, to be taken twice a-day, in a little sugar and water. By the time she had taken five powders, the disease left her.

Case IV.—August the 10th. Mr. P—'s son had been affected with the disease of the quotidian form for several days. I gave him a dose of the sub. mur. hyd.; and, after its operation had ceased, directed one powder of the prussiate of iron to be given in a small quantity of milk every three hours during the apyrexiæ. He had taken but four of the powders, when he became perfectly relieved, and remained in the enjoyment of health for some time.

Case V.—August 17th. I left Mr. P—'s child (who was labouring under feb. interm. and whose bowels had been freely evacuated, and had been taking the barks in considerable quantities, but to no effect,) several powders of the prussiate of iron, and directed one to be taken morning and evening, in sugar and water. It took them all without any benefit resulting from their administration. From this case it will be perceived that neither the bark nor prussiate of iron had any effect upon the disease.

Case VI.—August 19. Visited Mr. J—n's child, which I found affected with feb. intermittens, and to which I ordered a small dose of the pulv. rad. rhœi to be taken in a small quantum of syrup. simp., and which was succeeded by the internal use of six powders of the article that is the subject of this paper, but without any visible effects.

CASE VII.—Sept. 10. This day saw Mr. P—e's child; I gave it a dose of the sub. mur. hyd., which operated freely; after which I directed one powder of prussiate of iron to be given morning and evening. The child had taken but four powders when it obtained relief.

CASE VIII.—Sept. 16. Mr. H—y had two children who were for some considerable time labouring under the influence of this distressing malady. I left both of them cathartic medicines, which operated satisfactorily; I afterwards left each of them eight powders of the prussiate of iron. One of them became perfectly relieved, and the disease in the other case merely assumed the postponing form.

CASE IX.— Miss D—e I was called upon to visit about the middle of September. Upon paying my first visit, she appeared to be but triflingly indisposed; being apprehensive of her suffering from an attack of the then prevailing epidemic, I prescribed an emetic, which was succeeded by a dose of calomel. Upon my second visit, I found her unfortunately much worse, for by this time she was labouring under a high grade of bilious intermittent fever. I kept her bowels free, and during the apyrexia ordered a teaspoonful of the best barks to be given every three hours, which she continued to use for four days, without experiencing any benefit. After this I concluded to give my new remedy a trial: she commenced its use, and was after this saved from suffering the recurrence of another paroxysm.

CASE X.—Mr. B—h had been sick, during the course of the summer, of feb. intermittens. This gentleman had taken emetics repeatedly, succeeded by the use of cathartics of various kinds; he had likewise been in the constant habit of using barks in large quantities; and, as he observed, "he had taken almost every thing he had heard of, and that had been recommended to him," without his experiencing any alleviation. I left him four powders of the prussiate of iron, each containing six grains, and ordered one to be taken morning and evening; when he became perfectly relieved, and has never since had a return of the disease.

CASE XI.—Mr. J—'s son, aged fifteen years, was sick of intermitting fever four weeks. I gave him an emetic of euphorbia corollata, (or coralloid spurge,) which was followed by a dose of sub. mur. hyd. cum pulv. jallap; afterward I ordered one powder of the prussiate of iron to be given, morning, noon, and at night. This case recovered after having taken but six doses.

A very great number of cases more might have been added to the list, but it is deemed entirely superfluous at this time to occupy the reader's attention, inasmuch as the successful use of the remedy under consideration has so far been sufficiently established, from the cases already cited. I have merely to observe, that at the present period I am in the constant habit of using this article, in preference to the powdered barks, and find it to possess very decided advantages over this celebrated remedy.—(*American Medical Repository*, July 1822.)

**Art. III.**—*Account of a Case in which an Artificial Joint was cured by the Introduction of a Seton.* By Dr. H. W. Ducachet, New-York.

On the 20th December, 1820, Mr. E. G., of Pompton, New Jersey, was thrown from his waggon, and had the bones of his left fore-arm broken by the wheels passing obliquely across it about half-way between the elbow and the hand. Partly from the injudicious treatment of his attending surgeon, and partly, perhaps, from his own improper interference, the limb was neglected for a considerable length of time. After consulting a number of the medical men of the neighbourhood, it was ascertained that an artificial joint had formed; and a variety of unsuccessful means having been resorted to, he was advised to come to New-York for professional assistance. He applied to me on the 18th September 1821, and was encouraged to submit to the introduction of a seton between the fractured extremities of both bones. Unwilling, however, to believe that the ulna was not united, he would consent to the operation upon the radius only. Accordingly, on Wednesday, the 24th of October, 1821, with the assistance of my friend Dr. J. Kent Platt, and after making the necessary incision, I passed a suitable needle armed with a skein of silk between the ends of the bones. I dressed the wound in the usual manner, with adhesive straps, lint, &c., and, putting the arm into a sling, permitted him to return home. In about seventeen or eighteen days, it began to inflame and become painful, and in about three days more it discharged freely. On the twenty-fifth day after the operation, he came to see me. I found the limb so firm as to convince me that a bony union had commenced, and I accordingly applied the splints in the manner pursued in fractures of both bones of the fore-arm. In about six weeks after the operation I saw him again, and found the limb so strong and immovable that I removed the seton, notwithstanding the received direction that it should be suffered to remain at least twelve weeks. In two or three days he left off the splints and bandages, and in a week the wound was entirely healed. He gradually recovered the use of his arm; and in a letter to me, bearing date 1st June, 1822, he states that it has so far recovered its strength, that he has loaded and unloaded a waggon of hay without inconvenience. As soon as his business will permit him to come to town, I purpose to pass a seton through the artificial joint in the ulna. His incredulity as to the existence of this condition of the ulna prevented me from performing the operation at first, and his subsequent necessary detention at home has delayed it to this late period. I trust the plan will prove completely successful, and thus furnish additional evidence of the original genius and admirable practical sagacity of its inventor, and convince our transatlantic brethren that American surgery has not been altogether unfruitful in improvement. Before this happy idea was advanced by Dr. Physick, the unfortunate subject of an artificial joint was forced either to lose the use of his limb, or to undergo precarious and painful operations, which might be not only unproductive of advantage but even attended with danger, and which, at best, were succeeded by deformity: but now, by means of a simple operation, requiring only a few minutes for its performance, he may in a few weeks regain its strength as completely as before the accident.—(*Amer. Med. Rec.* July 1822.)

